# Arguments for the Adoption of a Nystagmus Care Pathway

**DOI:** 10.22599/bioj.126

**Published:** 2019-04-25

**Authors:** Christopher M. Harris, Julie Owen, John Sanders

**Affiliations:** 1 Orthoptic Department, Royal Eye Infirmary, Plymouth, UK; 2 Nystagmus Network, Kent, UK; 3 Psychology Department, Plymouth University, Plymouth, UK

**Keywords:** Nystagmus, Care Pathway, Nystagmus Network, Orthoptists, eye movement recording

## Abstract

Pathological nystagmus is a spontaneous oscillation of the eyes. It is a complex problem with many subtypes and causes ranging from the acute neurological emergency to chronic visual disorders. There is considerable variability in clinical management and patient experience across the UK. The Nystagmus Care Pathway (NCP) is a proposal to provide an evidence-based, consistent minimum standard of care across all eye services for patients with nystagmus. The NCP coordinates expertise from the various team members with a staged approach: 1) pathway entry; 2) nystagmus identification; 3) finding underlying causes/associations; 4) managing causes/associations; 5) managing the nystagmus and its effects; 6) support for patients and families; 7) pathway exit. Orthoptists are ideally placed to coordinate the NCP as they are trained in ocular motility and visual assessment. They are accustomed to providing continuity of care, multidisciplinary working and via the British and Irish Orthoptic Society (BIOS), they can provide consistency of care across the UK. Key performance indicators are proposed.

## Introduction

Pathological nystagmus is a difficult problem to manage. Onset can be at any age from early infancy onwards. There are many types of nystagmus that can be signs of underlying conditions ranging from the so-called ‘acquired nystagmus’ caused by acute or sub-acute neurological insults with adult onset to the chronic developmental (‘early-onset nystagmus’) with onset in infancy including infantile nystagmus syndrome (INS), formerly known as congenital nystagmus, and fusional maldevelopment nystagmus syndrome (FMNS), formerly known as manifest/latent nystagmus (Table 1) (Leigh & Zee 2015). However, neurological types of nystagmus can occur in infancy and early childhood and adults can present with a previously unidentified developmental nystagmus. Often the underlying cause or association cannot be identified and the nystagmus is labelled as ‘idiopathic’. Thus, the differential diagnoses of a patient presenting with nystagmus at an eye clinic are very broad. In the UK, there are six specialist nystagmus centres with eye tracking facilities, but nystagmus is not a rare condition. The prevalence for all types and all ages is 2.4 per 1,000 people (Sarvananthan et al. 2009), which amounts to about 160,000 people with nystagmus in the UK — far beyond the capacity of a few centres.

**Table 1:** Types of nystagmus.

Type of Nystagmus	Comment

**Infantile Nystagmus Syndrome (INS)**	• Formerly Congenital Nystagmus. May also be periodic alternating
**Fusion Maldevelopment Nystagmus (FMNS)**	• Formerly Latent nystagmus
**Gaze evoked (GEN)**	†
**Acquired Pendular (APN)**	†
**Periodic Alternating (PAN)**	†
**Downbeat (DBN)**	†
**Upbeat (UBN)**	†
**Torsional**	†
**Vestibular**	† Central or peripheral
**See-Saw**	†
**Epileptic**	† Ictal nystagmus
**Ocular flutter/opsoclonus**	† back-to-back saccades (not strictly nystagmus)
	• - early onset † - ‘acquired’ but may occur in early childhood due to CNS (non-retinal) abnormality.

## Clinical Perspective

Patients with nystagmus are usually referred to their local eye clinic for further investigations. There is, however, no standard flow-chart or accepted strategy for investigation. Older patients with late onset nystagmus and obvious neurological signs require neurological investigation and brain imaging. Patients with early onset of nystagmus require ophthalmological investigation in the first instance (CEMAS Working Group 2000). However, many patients are not so clear-cut at presentation often due to vague history. Not surprisingly, therefore, there are wide variations in clinical practice. This variability is compounded by out of date practices and a lack of modern equipment. Thus, in the 1970s and 1980s numerous studies reported eye movement recordings of all types of nystagmus. The seminal work of Dell’Osso & Daroff (1975) showed that INS, specifically, could be positively identified and distinguished from acquired nystagmus. It afforded an objective way to identify a patient’s nystagmus by its waveform and, crucially, not by the underlying association. This meant that INS could be identified at any age from infancy to adulthood and that neurological investigations could usually be avoided. It also showed that what appeared from simple observation to be pendular nystagmus usually turned out to be jerk nystagmus, thus dispelling the longstanding heuristic that pendular implied sensory defect and jerk nystagmus implied idiopathic early-onset nystagmus (Allen & Davies 1983). Unfortunately, eye tracking and waveform analyses are still luxuries found only in a few specialist centres.

Even with eye tracking facilities, the problem of finding underlying visual sensory defects in INS remains. Prior to the 1990s most cases of INS were diagnosed with albinism or idiopathic (‘motor’) nystagmus based on the ophthalmoscope and the slit lamp. By the 1990s, however, the introduction of electrodiagnostics based on visual evoked potentials (VEPs) and the electroretinogram (ERG) and eye movement recording at Great Ormond Street Hospital revealed that many presumed INS idiopaths exhibited a range of developmental sensory defects and that albinism was under-diagnosed (Kriss & Russell Eggitt 1992; Taylor 1997). Today, the usefulness of electrodiagnostics is widely recognised but still only available in some hospitals.

The last decade has seen a surge in the use of optical coherence tomography (OCT) to image the retina in nystagmus patients, and has revealed previously undetected abnormal macular morphology, especially foveal hypoplasia. Some patients, once considered to be idiopathic, may now be found to have abnormal retinal morphology, which is now routinely graded according to morphological features (Thomas et al. 2014). For many years X-linked pedigrees with INS idiopathic nystagmus have been identified and the discovery of the *FRMD7* gene was heralded as a breakthrough in understanding idiopathic INS (Tarpey et al. 2006). A recent discovery is that patients with *FRMD7* mutations also have subtle foveal hypoplasia and optic nerve head abnormalities (Thomas et al. 2014) raising doubts about the ‘idiopathic’ label. Foveal hypoplasia is also associated with optic nerve hypoplasia in INS (Pilat et al. 2015). Thus, OCT is now an essential tool in the investigator’s armoury and is available in all eye centres, but is still difficult to use in the young. The hand-held OCT is clearly more successful (Lee et al. 2013) but currently only available in a few centres.

Although, at time of writing, six specialised nystagmus services have emerged in the UK, many young children and adults are still not thoroughly investigated, as can be clearly seen from the feedback reaching Nystagmus Network.

## Patient Perspective

The Nystagmus Network (NN), previously called nystagmus action group, was set up in 1984 as a support group for people with nystagmus. For 30 years, it has received a steady flow of feedback from patients and parents via letters, emails, phone calls, and social media. Audit data for enquiries to the charity’s helpline for 2015 showed a total of 1,186 enquiries in 2015, equivalent to more than three per day (Table 2).

**Table 2:** Total enquiries (phone, email and social media) to Nystagmus Network helpline 2015. Audit data prepared for NN annual report 2015 and presented at NN AGM, Birmingham, 7th May 2016.

Category	No. Enquiries

UK general public enquiries	495
Overseas enquiries	127
Enquiries from UK professionals	271
Administration, fundraising, volunteering	293
TOTAL	1186
UK general public enquiries by category	

General support & information	233
Research & treatment	70
Education	64
Benefits & discrimination	47
Acquired nystagmus	29
Employment	18
Driving & transport	18
Other	16
Sub Total	495

Many of these enquiries were requests for basic information about nystagmus and its impact. For instance, parents often asked whether their children see the world moving, why they adopt an abnormal head posture, and what help is available at school. Many were not aware of the null zone, the Certificate of Vision Impairment (CVI), or that their child could be supported by a Qualified Teacher of the Visually Impaired (QTVI). At the point of discharge from the hospital service, some parents had not been warned that their child is unlikely to be able to drive, or conversely, have been told that they will never drive, when their visual function is actually sufficient to pass the driving standard. NN also hears from parents every year who have been told that there is nothing wrong with their child’s eyes. While in the strictest sense this may sometimes be true, it is highly misleading as most parents then incorrectly assume there is nothing wrong with their child’s vision.

It is difficult to assess the overall number of patients and families that do not receive basic support and information, but NN data shows hundreds every year. As a result, these patients are at risk of performing poorly at school, being unemployed, or becoming depressed and isolated. Anecdotal evidence collected by NN suggests strongly that this is a worldwide problem.

There has been surprisingly little study of the quality of life (QOL) of people with nystagmus. In a postal questionnaire of NN members with nystagmus, Pilling et al. (2005) found a strong correlation between visual function and social function. McLean et al. (2012) carried out a QOL study on 21 patients with nystagmus using semi-structured interviews. They identified 6 domains that were adversely affected by nystagmus: visual function, movement restriction, standing out or not fitting in, feelings about inner self, negativity towards the future, and relationships, each with various subcategories. All participants reported that nystagmus affected every aspect of everyday life and some participants were visibly distressed when interviewed. These overwhelmingly negative effects cannot be attributed solely to the impact of low VA. Other significant, but underestimated, visual consequences exist and are rarely measured. Furthermore, the cosmetic effect of nystagmus was clearly important and the authors drew parallels to similar issues with strabismic patients including issues with self-image, relationships, and self-esteem (Archer et al. 2005). One can only conclude that nystagmus has a negative impact on life.

Information gathered by NN shows a disconnection between what patients and their families expect, and what clinical teams deliver. Some centres provide exhaustive investigations, treatment where possible, and discuss the nystagmus with patients and their families at length. Others do not. The reason for these differences cannot just be blamed on time and money, although these are not insignificant issues; other factors to include training, experience, and attitudes towards nystagmus also need to be considered.

## Proposed Nystagmus Care Pathway (NCP)

We, the authors, propose that the NCP would have the following advantages:

- The NCP outlines a minimum standard of care across all centres regardless of the actual hospital or medical specialists.
- The NCP is a multi-disciplinary approach that is patient-centred and recognises nystagmus as a condition in itself as well as an association of underlying pathology.
- The NCP has been developed from current evidence-based practice and so outlines a recommended, standardised approach for all patients with nystagmus.
- The NCP offers the possibility of cost reductions by minimising the number of follow-up visits with no clear purpose. More cross-referrals may be needed, but diagnosis will be made more quickly. This, and the proposed key performance indicators (KPIs) with subsequent audit opportunities have clear benefits both for patients and their healthcare providers.
- The NCP can evolve over time as new evidence-based developments occur.
- The NCP is an easy-to-follow staged approach.

We identify seven stages to the NCP:

1. Pathway Entry
2. Nystagmus Identification
3. Finding Underlying Causes/Associations
4. Managing Underlying Causes/Associations
5. Managing the Nystagmus and its effects
6. Support for patients and families
7. Pathway Exit

Adhering to these stages clarifies and organises their separate objectives and will usually involve ophthalmologist, orthoptists, optometrists, clinical scientists, Qualified Teachers for Sensory Support (QTSS or QTVI), and Rehabilitation Officers for Visual Impairment (ROVIC). The importance of each stage will vary across patients, but we advise that all stages be followed so that especially stages five and six are not overlooked. The sequence may vary depending on the patient presentation. We now consider each stage.

### Pathway Entry

All patients with nystagmus should enter the pathway. Nystagmus may not be the presenting sign and the patient may be under care for an associated condition or for multiple disabilities. This also includes children attending special schools (Woodhouse et al. 2013). We suggest a NCP manager, such as an orthoptist, coordinates the entry, progression, and exit of patients in the pathway.

### Nystagmus Characterisation

An **explicit** effort should be made to characterise and, where possible, identify the type of nystagmus. This often narrows down likely underlying conditions and guides subsequent stages. Eye movement recording provides a huge advantage over clinical observation, as the waveform can be objectively visualised. Identifying an INS waveform can be crucial because if no underlying visual cause can be found in the next stage, it usually obviates the need for neuroimaging. Without this positive identification, one can never be absolutely sure that the nystagmus is not neurological. If eye movement recording is not available, the use of the diagrammatic representation of ocular movements in the nine cardinal positions can be useful. However, such diagrams must be able to depict torsional(rotary) and circumrotatory nystagmus’s, as well as the more common jerk and pendular types, including combinations of more than one type (Osborne et al. 2019). It should be recognised that these diagrams rarely identify the type of nystagmus. Referral to a centre with eye movement recording facilities would be preferable. If the nystagmus cannot be identified, or there is some ambiguity/other eye movement abnormality, this should be **explicitly** stated in the medical records to avoid any false assumptions that the nystagmus has been identified. Indeed, failure to identify the type of nystagmus with eye movement recording may be an important clue to the underlying cause.

### Investigations of the Underlying Causes/Associations

In most cases, there is an underlying association or cause of the nystagmus. Associations are varied and well recognised in the clinical literature (not reviewed here). For the most part, however, precisely why nystagmus emerges is poorly understood. For example, INS is often associated with abnormal foveal development or other interference with macular/foveal vision from birth (e.g. cataracts). But why these actually lead to the development of nystagmus is not known. On the other hand, infantile epileptic nystagmus is almost certainly caused by focal hyperactivity in the oculomotor pathways (Harris et al. 1997), but the cause of the epilepsy may be cryptic. Regardless of what we mean by ‘cause’, nystagmus is a sign of underlying neurological or ophthalmological pathology and it is a clinical priority to find it.

Most types of nystagmus will fall into one of two broad categories (Casteels et al. 1992):

- Early-onset nystagmus (<6 months), which includes Infantile Nystagmus Syndrome (INS), formerly congenital nystagmus, and Fusion Maldevelopment Nystagmus (FMNS), formerly known as latent nystagmus. The majority of associations are sensory (see Table 3 for typical associations) and, hence, require ophthalmological investigations in the first instance.
- Late-onset or ‘acquired’ (>6 months), and includes many types of nystagmus. The majority of cases have an underlying neurological cause, rather than sensory, and require neurological assessment.

**Table 3:** Some typical sensory defects associated with early-onset nystagmus.

Infantile Nystagmus Syndrome

Achromatopsia
Albinism (oculocutaneous, ocular)
Aniridia
Cataracts (bilateral)
Colobomata
Cone dysfunction
Congenital Stationary Night Blindness
Foveal Hypoplasia (isolated)
Optic Nerve Hypoplasia (bilateral)
PAX6 mutations
Fusion Maldevelopment Nystagmus

Infantile esotropia
Cataract (unilateral)
Optic Nerve Hypoplasia (unilateral)

The categories in Table 1 are only a guide. Some infants and young children will have ‘acquired’ nystagmus with an underlying neurological cause. Some adults will have previously undiagnosed INS or FMNS or more rarely nystagmus secondary to acquired visual loss. Eye movement recording from stage 2 is therefore very useful, since INS can be positively identified at any age, and acquired nystagmus can usually be categorised.

From an eye clinic perspective, the minimum standard should include the list below (many of these entries are standard):

- Family history as all types of nystagmus are sometimes inherited.
- Patient history including any neurological history.
- Orthoptic examination.
- Ophthalmological examination.
- OCT.
- Referral for electrodiagnostics including age appropriate ISCEV standard ERG and VEPs should be made if no underlying explanation for the nystagmus is found. In particular, the label of ‘idiopathic nystagmus’ cannot be applied until electrodiagnostic investigations have been carried out satisfactorily and reveal no underlying cause.
- If the nystagmus waveform is not positively identified as INS or FMNS: neurological examination and MRI of brain – refer to neurology (appropriate for age). Note: if INS or FMNS have been positively identified, brain imaging is not usually needed unless neurological associations are suspected (e.g. septo-optic dysplasia).
- Genetic testing for patients with nystagmus is not widely available on the NHS. Specific tests are available for specific diseases where there is a very high level of suspicion such as a retinal dystrophy gene panel (£897) or *FRMD7* gene sequencing (£400) in cases of typical INS with a strong X-linked family history.

### Management of the Underlying Cause/Association

There is a huge range of pathological conditions associated with nystagmus and each requires specific management according to standard practice. This stage may be a dominant and long-term aspect of patient care. Although such management may modify the nystagmus, the nystagmus very often persists. Thus, we believe it is important to separate the management of the association from that of the nystagmus.

Neurological associations are usually managed by the neurology team and may include treatments for tumours, demyelinating disease, trauma, and vestibular disorders. Even when brain lesions can be identified there is often no realistic treatment.

Associated visual disorders and strabismus will usually be managed by the eye clinic. For most cases, the underlying visual disorder will have a congenital or perinatal origin. For many conditions there are no treatment options. Even when treatment is available in infancy (e.g. cataract surgery), evidence indicates that the nystagmus (INS or LN) often persists (Young et al. 2012).

### Managing the Nystagmus

The assumption is that the nystagmus is persistent at this stage. The objective is then to manage the nystagmus and its consequences. This includes:

- treatment of nystagmus & its effects where possible
- support for psychological, educational, and social aspects

#### Oscillopsia

Oscillopsia is the illusion that objects are oscillating. It is a complex perceptual disorder that is difficult to manage in people with nystagmus (Straube et al. 2011). In acquired nystagmus a variety of drug therapies has been described with variable success (Mehta & Kennard 2012). Currently, the drug of choice depends on the type of nystagmus, such as downbeat, upbeat, acquired pendular, periodic alternating, and epileptic nystagmus.

In INS, oscillopsia is not a presenting sign and seldom bothersome. Nevertheless, it is not uncommon for patients to report illusion movement when their nystagmus is more intense than usual (stress, concurrent illness, far from null).

#### VA

For INS, current evidence indicates that the nystagmus *per se* has only a minor contribution to reduced VA, at least in adults (Dunn et al. 2014; Dunn et al. 2017). Poor VA either reflects the underlying associated condition, known or unknown, and/or amblyopia secondary to the nystagmus. Treatments aimed at reducing nystagmus intensity have only small effects on VA — even though they are often successful in reducing the nystagmus intensity itself for some patients.

Abnormal head postures can sometimes be managed by prismatic correction or surgery. Suitable patients should be referred to optometrists or pphthalmologists for optical or surgical treatment as required. Debate still exists regarding any additional benefits gained from extra-ocular muscle surgery besides correcting squints and head posture. Until this is reconciled, we would advocate that extra-ocular muscle surgery is performed for squint and head postures alone and consider additional possible benefits, such as described widening of the null zone and reduction in nystagmus intensity as possible, but as yet unproven.

### Support

There are numerous aspects to supporting patients and their families with nystagmus. It is important to recognise that many useful supports exist (governmental, educational, charities), but how well patients or parents engage with or navigate through such agencies varies considerably. A key to unlocking support is to recognise that nystagmus is a ‘disability’ in itself rather than a just a sign of some underlying pathology. Some issues that need to be broached are:

- A frank discussion about nystagmus with patient and family including causes, mechanisms, and prognosis.
- Explain the functional consequences of nystagmus beyond poor VA, including effects on activities of daily living (ADLs), time-to-see, effects of stress, crowded and unfamiliar visual environments, education, etc.
- It is important to clearly state that the nystagmus is likely to be life-long, and that realistic treatments for improving vision are limited.
- Patients with nystagmus are not alone in having their condition and should be given a Nystagmus Information pack (https://www.sheffield.ac.uk/oncology-metabolism/research/ophthalmology-orthoptics/research/nystagmusinfo) and directed to national organisations such as the Nystagmus Network, RNIB, Albinism Fellowship, Aniridia Network and to local organisations such as Useful Vision (north-east), Angel Eyes, (Northern Ireland), Moorvision (Devon), IN-Vision.
- Consider referral of patient and family for counselling if they are overwhelmed by the nystagmus and/or visual impairment. Referral to the nearest rehabilitation officers for visually impaired children (ROVIC) may be an option.
- Certificate of Vision Impairment (CVI) and the benefits of certification and registration. In practice, various benefits are more likely to become available with CVI registration although we are not aware of any statutory linkage. CVI should be considered for patients even with reasonably good VA because of other significant visual difficulties including oscillopsia, fatigability, effects of stress and poor lighting on vision, long visual response times, difficulty with motion and stereopsis, and issues with a null region and/or any compensatory head posture.
- Genetics. Acquired and early-onset nystagmus may be inherited irrespective of any known family history. Genetic counselling and clinical genetics referral should be offered.
- Education: pre-school and school-aged children should be referred to local advisory qualified teachers for vision impairment (QTVIs), but it is important to explain that teachers may not be familiar with nystagmus.
- Driving: note that nystagmus is a ‘known medical condition’ and must be declared to the DVLA (gov.uka 2016).
- Benefits (gov.ukb 2016):
  - Disability Living Allowance (DLA) and the Personal Independent Payments (PIP).
  - Employment Support Allowance (ESA).
  - ‘Attendance Allowance’ for pensioners.
  - There is also now the introduction of Universal Credit, which is still in its infancy.

### Pathway Exit

In our experience, patients are often followed up for years. The reason for this is not clear. The nystagmus itself does not change much in most cases. Progressive visual disorder or acute neurology should have been diagnosed, so there is most commonly no visual deterioration to expect. Refractive errors may change, but this in itself does not warrant eye clinic visits once the child has reached an age when optometrists in the community can refract them. Presumably, follow-up visits are an insurance plan in case something subsequently goes amiss. However, we are not aware of any evidence base to justify this *provided* the previous stages have been fulfilled.

The costs, on the other hand, are more tangible. In over-stretched clinics, filling NHS slots with follow-up visits is a substantial workload. From the patient’s perspective, time off work and/or school and travelling to and from a clinic is not trivial for some. There is also the important issue that longitudinal visits unnecessarily maintain a high level of patient medicalisation and unfulfilled expectations.

We suggest that patients should be discharged from the pathway when there is no tangible benefit for continued follow-up. Indeed, having a pathway should make such discharge possible. Thus, when the nystagmus has been identified, underlying pathology has been positively identified, or all currently available investigations have been exhausted, treatments, if any, have concluded, and accurate linear LogMar VA achieved then it is feasible to discharge. However, it is crucial that the following discharge standards have been met:

- NCP is exhausted and referrals completed.
- Contact with the patient is maintained so that they can return at some later date, if desired.
- Full information is provided at discharge. As discussed above, lack of information is a major source of complaint.

Refractive and visual check-ups are available outside the eye clinic by opticians/optometrists who have a full understanding of nystagmus.

## Outcome Measures & Future Adaptations

### Key Performance Indicators

A key requirement of the NCP is the need to track patients through the pathway. We propose that the NCP is facilitated by a database and since there are no published guidelines at present, this will provide key performance indicators (KPIs). These have been agreed to in principal by the Professional Development Committee of BIOS and include the following:

- caseloads and breakdown of nystagmus types
- evidence of clinical protocols including statistics effectiveness of
  - ocular investigations
  - electrodiagnostics
  - OCT
  - MRI
  - support services
  - discharges

There would be an obvious advantage of maintaining the same database structure across eye clinics, as this would provide a national epidemiological database.

### Future Adaptations

In our view, a NCP needs to adapt to innovation and developments in medical science. OCT is under continuous development, and hopefully the ease of use with young children will improve, and age related norms will emerge. It is likely that genetic testing will become routine in the next few years as costs tumble and genetic panels for nystagmus become available. Indeed, it seems feasible that testing for a bank of known nystagmus genes or even sequencing all of a patient’s genes will become the primary investigation. Early-onset nystagmus means a lifetime of visual impairment. VA is an inappropriate measure of functional vision for nystagmus. There is now a strong move to look for ‘time-to-see’ measures, as these seem more sensitive to waveform manipulations. Whether these will be fruitful, and whether they can be translated into the clinic, remains to be seen. But if there is an evidence-base, then it should be adopted by the NCP in the future.

## Summary

According to Google Scholar, there have been over 3,000 publications on nystagmus since 1991 (search word ‘nystagmus’ in the title). Yet, in our collective experience, including experts in the specialist clinical centres in the UK, the investigation of patients with nystagmus has changed little in *most* eye departments. The charity Nystagmus Network was formed in 1984 by patients and their families in response to a lack of information, wildly different experiences across centres, and a perceived lack of interest by clinicians. Judging from the flow of complaints the disconnection between what clinical teams believe they provide, and what patients and families believe they receive, has not gone away. We propose that a NCP would help close this gap by providing a consistent and minimum standard of care across all centres.
